# Potential Impact of ALKBH5 and YTHDF1 on Tumor Immunity in Colon Adenocarcinoma

**DOI:** 10.3389/fonc.2021.670490

**Published:** 2021-05-17

**Authors:** Guanyu Yan, Yue An, Boyang Xu, Ningning Wang, Xuren Sun, Mingjun Sun

**Affiliations:** Department of Gastroenterology, The First Hospital of China Medical University, Shenyang, China

**Keywords:** colon adenocarcinoma, ALKBH5, YTHDF1, immune contexture, m6A modification

## Abstract

**Background:**

ALKBH5 and YTHDF1 are regarded as the eraser and reader, respectively, in N6-methyladenosine (m6A) modification. Recently, immune contexture has been drawing increasing attention in terms of the progression and treatment of cancers. This study aimed to determine the relationship between ALKBH5/YTHDF1 and immunological characteristics of colon adenocarcinoma (COAD).

**Methods:**

Expression of ALKBH5 and YTHDF1 was investigated across TCGA and GEO validated in our study. Patients with COAD were divided into two clusters using consensus clustering based on the expression of ALKBH5 and YTHDF1. We then compared their clinical characteristics and performed gene set enrichment analysis (GSEA) to identify the functional differences. Immune infiltration analyses were conducted using ESTIMATE, CIBERSORT, and ssGSEA. In addition, we evaluated the expression of the targets of immune checkpoint inhibitors (ICIs) and calculated the tumor mutation burden (TMB) of the tumor samples. Weighted gene co-expression network analysis (WGCNA) was used to identify the genes related to both ALKBH5/YTHDF1 expression and immunity. GSE39582 was utilized for external validation of immunological features between the two clusters.

**Results:**

Cluster 2 had high expression of ALKBH5 and lesser so of YTHDF1, whereas Cluster 1 had just the reverse. Cluster 1 had a higher N stage and pathological stage than Cluster 2. The latter had stronger immune infiltration, higher expression of targets of ICIs, more TMB, and a larger proportion of deficiency in mismatch repair-microsatellite instability-high (dMMR-MSI-H) status than Cluster 1. Moreover, WGCNA revealed 14 genes, including PD1 and LAG3, related to both the expression of ALKBH5/YTHDF1 and immune scores.

**Conclusions:**

ALKBH5 and YTHDF1 influence immune contexture and can potentially transform cold tumors into hot tumors in patients with COAD.

## Introduction

Colorectal cancer (CRC) ranks third in incidence, and its mortality ranks second in both sexes worldwide (1). Approximately 70–80% of patients with early stage CRC are eligible for surgery, and the five-year survival rate of these patients is approximately 90%. However, the five-year survival rate of patients with distant CRC (stage IV) is as low as 10–15% (2, 3).

Colon adenocarcinoma (COAD) is the main type of CRC that originates from adenomatous lesions and evolves into cancer due to the accumulation of genetic mutations (4). Recently, a study has shown that mutations can generate new antigens, which can be recognized by the immune system (5). Moreover, immunotherapy has proven to be effective in treating advanced carcinomas (6). Nevertheless, immunotherapy has limitations in some patients with microsatellite instability (MSI) and most patients with microsatellite stability (MSS) (7). Therefore, identification of novel immunotherapy markers and uncovering the underlying mechanisms of immune checkpoints would be important.

N6-methyladenosine (m6A) is the most abundant RNA modification that occurs in both coding and non-coding RNAs, and is a crucial post-transcriptional regulator in various cancers (8–11). Proteins involved in m6A modification may be divided into three categories: writer (catalyzes the occurrence of m6A modification), eraser (catalyzes the removal of m6A modification), and reader (recognizes and binds m6A modification) (12). Although tumor-intrinsic carcinogenic processes are vital, the impact of m6A modification on tumor and immunity is also worth attention. In recent years, some studies have suggested that targeting of dysregulated m6A regulators with small molecule inhibitors has potential in treating cancer. Given the functional importance of m6A modification in various cancers, targeted treatment against m6A regulators may be applicable in the clinic, in combination with chemotherapy or immunotherapy, to improve cancer therapy (13).

ALKBH5 plays the role of eraser in m6A modification and has been proven to regulate suppressive immune cell accumulation in melanoma (14, 15). In pancreatic ductal adenocarcinoma, ALKBH5 inhibits tumorigenesis by decreasing m6A modification of WIF-1 RNA and mediating the Wnt signaling pathway (16). On the other hand, YTHDF1 is a reader in m6A modification that can improve the efficiency of mRNA translation (17). It regulates the expression of lysosomal proteases in an m6A-dependent manner to control anti-tumor immunity and improve the efficacy of immunotherapy. Deficiency of YTHDF1 can enhance the therapeutic effect of PD-L1 checkpoint blockade (18).

To comprehensively understand the role of ALKBH5 and YTHDF1 in tumor immunity, we analyzed the transcriptome profiling data of colon adenocarcinoma from The Cancer Genome Atlas (TCGA) and Gene Expression Omnibus (GEO). We divided patients into two clusters based on the expression of ALKBH5 and YTHDF1 and compared their differences in clinical characteristics, biological pathways, immune infiltration, immune checkpoint expressions, and mutational landscapes using bioinformatics methods. Our results indicated that Cluster 2, with high expression of ALKBH5 and low expression of YTHDF1, had more immune infiltration, immune checkpoint inhibitor expression, and tumor mutation burden than Cluster 1, hence suggesting that Cluster 2 might respond better to immunotherapy. ALKBH5 and YTHDF1 may remarkably influence the immune contexture of colon adenocarcinoma.

## Materials and Methods

### Data Sources and Preprocessing

Transcriptome profiling data (HTSeq-Counts and HTSeq-FPKM) with clinical information were downloaded from TCGA-COAD project by R (version 4.0.2) with R package TCGAbiolinks (19). Cases that contained intact clinical information (age, sex, T stage, N stage, M stage, and prognostic information) were included. Level 3 HTSeq-FPKM of 435 primary solid tumor samples were treated by log2(FPKM+1) transformation for further analyses, and HTSeq-Counts were used for differential analysis.

Simple nucleotide variation data (MuTect2) of 376 patients with COAD were collected using R package maftools (20). Due to the lack of mutation information for some patients with COAD, we only included 376 patients in the analysis involving mutational landscape. Waterfall plots were used to show the genetic mutation of patients using the R package ComplexHeatmap (21). Tumor mutation burden (TMB) was calculated based on simple nucleotide variation, defined as the number of mutations per megabase.

Expression profiling by GSE39582 array was downloaded from the Gene Expression Omnibus (GEO) database (https://www.ncbi.nlm.nih.gov/gds/) (22). The dataset with 566 colon cancer tissues was used to verify the immune characteristics of patients with colon cancer.

### Immune Infiltration Analysis

ESTIMATE is a method that determines the fractions of stromal and immune cells based on gene expression signatures in tumor samples. It was applied to evaluate the tumor microenvironment (TME) of each patient with COAD, along with stromal score (stromal content), immune score (extent of immune cell infiltration), ESTIMATE score (synthetic mark of stroma and immune), and tumor purity by R package estimate (23).

CIBERSORT is a means of computing cell composition based on the expression profiles. This deconvolution algorithm was used to calculate the proportion of 22 immune cells in each patient with COAD (24). The sum of 22 immune cell type fraction in each sample was 1.

By applying the single-sample gene set enrichment analysis (ssGSEA) method from R package GSVA (25), we calculated the extent of infiltration of 28 immune cell types according to the expression levels of genes in 28 published gene sets for immune cells (26).

### Consensus Clustering Based on ALKBH5 and YTHDF1

Expression of ALKBH5 and YTHDF1 was extracted and clustered coherently using the R package ConsensusClusterPlus (27). The samples were divided into two clusters. We used the R package CMScaller to identify the consensus molecular subtypes (CMS) of each sample (28). CMS is a robust classification system for CRC. Every CMS has distinct features: CMS1 (immune), CMS2 (canonical), CMS3 (metabolic), and CMS4 (mesenchymal) (29). A Sankey diagram was used to indicate the relationship between the two clusters and CMS.

### Gene Set Enrichment Analysis

GSEA was performed using the R package clusterProfiler to discover the significant functional difference between the two clusters (30). Significant pathway enrichment was identified by the normalized enrichment score (|NES| >1), P value <0.05, and FDR q value <0.05.

### Differential Expressed Genes

Expression profiling data (HTSeq-Counts) were compared to identify the DEGs of two clusters using the R package DESeq2 (31). The threshold values were |log2FoldChange | >1 and adjusted P value <0.05.

### Weighted Gene Co-Expression Network Analysis

We performed WGCNA on DEGs using the R package WGCNA (32). To ensure that the constructed co-expression network approached scale-free distribution, we chose 5 as the soft power. We obtained nine modules and calculated their correlation with cluster, stromal score, immune score, ESTIMATE score, and tumor purity. Subsequently, we acquired 14 genes according to the calculation of module membership (MM) and gene significance (GS).

### Functional Enrichment Analysis

Gene Ontology (GO) analysis was applied to understand the functions of 14 selected DEGs using the R package clusterProfiler (30). We then constructed a protein–protein interaction (PPI) network using the STRING database (33). Next, we analyzed the Spearman’s correlation of gene–gene, gene–ESTIMATE, and gene–ssGSEA using the R package corrplot.

### Specimen Collection and Real-Time Quantitative PCR

Twelve pairs of CRC tissues and their adjacent tissues were collected from the First Hospital of China Medical University with informed consent and approval from the Institutional Ethics Board of the First Hospital of China Medical University.

Total RNA was extracted using TRIzol reagent (Invitrogen). cDNA was synthesized using the PrimeScript RT Reagent Kit (TaKaRa). The SYBR Prime Script RT-PCR kit (TaKaRa) was used to perform RT-qPCR according to the manufacturer’s protocol.

The primer sequences were as follows: ALKBH5-F, 5′-CGGCGAAGGCTACACTTACG-3′; ALKLBH5-R, 5′-CCACCAGCTTTTGGATCACCA-3′; YTHDF1-F, 5′-ACCTGTCCAGCTATTACCCG-3′; YTHDF1-R, 5′-TGGTGAGGTATGGAATCGGAG-3′; GAPDH-F, 5′-CGGATTTGGTCGTATTGGG-3′; GAPDH-R, 5′-CTGGAAGATGGTGATGGGATT-3′.

### Statistical Analysis

All statistical analyses were conducted by R (4.0.2) and SPSS (25.0) software. Figure panels were pieced together by Adobe Illustrator (CC 2019). Box plot analyses were performed using the Wilcoxon rank-sum test. Correlation analysis was performed using the Spearman’s coefficient. Chi-square test was used to compare the clinical characteristics between the two clusters (Fisher’s exact test was used when required). Multivariate logistic regression analysis was used to evaluate the clinical characteristics affecting the clusters. Survival curves were constructed using the Kaplan–Meier method (log-rank test). All hypothetical tests were two-sided, and a P value <0.05 was considered significant.

## Results

### Identification of Immune-Related m6A Regulators and Consensus Clustering of Patients

**Figure 1:**
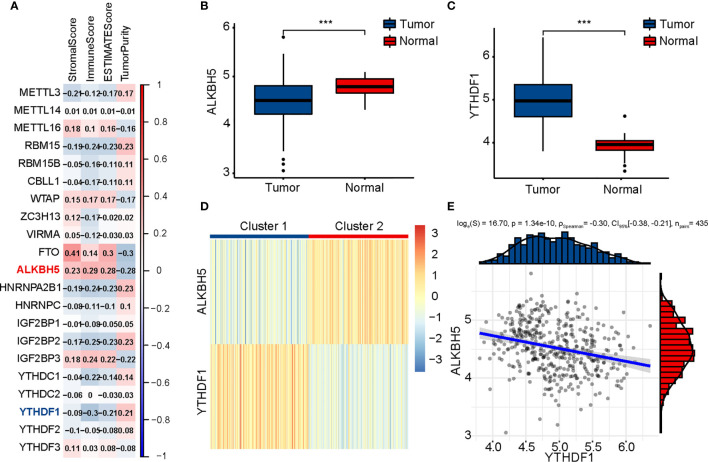
Identification of m6A regulators related to immune score and clustering of TCGA-COAD patients based on ALKBH5 and YTHDF1. **(A)** Association between m6A regulators and results of ESTIMATE. **(B)** Comparison of ALKBH5 expression between tumor and normal tissues. **(C)** Comparison of YTHDF1 expression between tumor and normal tissues. **(D)** TCGA-COAD patients are divided into two clusters according to ALKBH5 and YTHDF1. **(E)** Association between ALKBH5 and YTHDF1 expression. The P values are labeled using asterisks (***P < 0.001).

First, we calculated four indices of ESTIMATE in each sample to assess the fractions of stromal and immune cells. In order to explore the role of m6A modification in tumor immunity of patients with COAD, 21 m6A regulators were identified, and correlation between the expression of m6A regulators and results of ESTIMATE was evaluated (**Figure 1A**). Considering the highest absolute value of correlation with immune score, ALKBH5 and YTHDF1 were included in subsequent analyses. Next, Wilcoxon rank-sum test was conducted between tumor and normal tissues using RNA-seq data of TCGA-COAD; the tumor tissues were found to have lower ALKBH5 expression and higher YTHDF1 expression than normal tissues (**Figures 1B, C**). We thereafter performed a consensus clustering on 435 TCGA-COAD samples according to the expression matrix of ALKBH5 and YTHDF1, and divided the samples into two clusters (**Figure 1D** and **Supplementary Figure 1**). The heatmap shows Cluster 1 (n = 217) to have low expression of ALKBH5 and high expression of YTHDF1 while Cluster 2 (n = 218) had low expression of YTHDF1 and high expression of ALKBH5. Since the two genes showed opposite trends in the two clusters, Spearman’s correlation between ALKBH5 and YTHDF1 was investigated, and a weak, negative correlation (R = −0.30, P = 1.34e-10) was found (**Figure 1E**). To understand the features of the two clusters better, we evaluated the CMS of each sample and drew a Sankey diagram to indicate their relationship (**Supplementary Figure 2**).

### Evaluation of Clinical Characteristics

To identify the differences in clinical characteristics between the two clusters, we drew a survival curve first, and found no significant difference in prognosis between the two clusters (**Supplementary Figure 3**). Associated analysis showed Cluster 1 to have higher N stage, higher pathological stage, and lesser age than Cluster 2 (**Table 1**). Moreover, multivariate logistic regression analysis showed age, T stage, and N stage to be independent factors affecting clustering (**Table 2**).

**Table 1:** Clinical features of two clusters.

	Cluster 1	Cluster 2	P value
Number	217	218	
Age (median [IQR])	66.00 [56.00, 75.00]	71.00 [61.00, 80.00]	<0.001
Gender (%)			0.312
female	97 (44.7)	109 (50.0)	
male	120 (55.3)	109 (50.0)	
T stage (%)			0.42
T1	5 (2.3)	5 (2.3)	
T2	40 (18.4)	34 (15.6)	
T3	151 (69.6)	147 (67.4)	
T4	21 (9.7)	32 (14.7)	
N stage (%)			0.029
N0	114 (52.5)	141 (64.7)	
N1	60 (27.6)	41 (18.8)	
N2	43 (19.8)	36 (16.5)	
M stage (%)			0.098
M0	157 (80.5)	170 (87.2)	
M1	38 (19.5)	25 (12.8)	
Pathological stage (%)			0.033
Stage I	36 (16.6)	38 (17.4)	
Stage II	73 (33.6)	100 (45.9)	
Stage III	70 (32.3)	55 (25.2)	
Stage IV	38 (17.5)	25 (11.5)	

**Table 2:** Multivariate Logistic Regression for clustering (Cluster 2 *vs*. Cluster 1).

Variables	Multivariate Logistic Regression	
	Odds Ratio (95% Confidence Interval)	P value
age	1.02 (1.01–1.04)	0.0032
T stage		0.0705
T2 *vs*. T1	0.46 (0.11–1.91)	0.2828
T3 *vs*. T1	0.35 (0.16–0.77)	0.0088
T4 *vs*. T1	0.50 (0.27–0.93)	0.028
N stage		0.0129
N1 *vs*. N0	1.72 (0.99–2.98)	0.0525
N2 *vs*. N0	0.86 (0.47–1.58)	0.6212

### Identification of Immune-Related Pathways by GSEA

**Figure 2:**
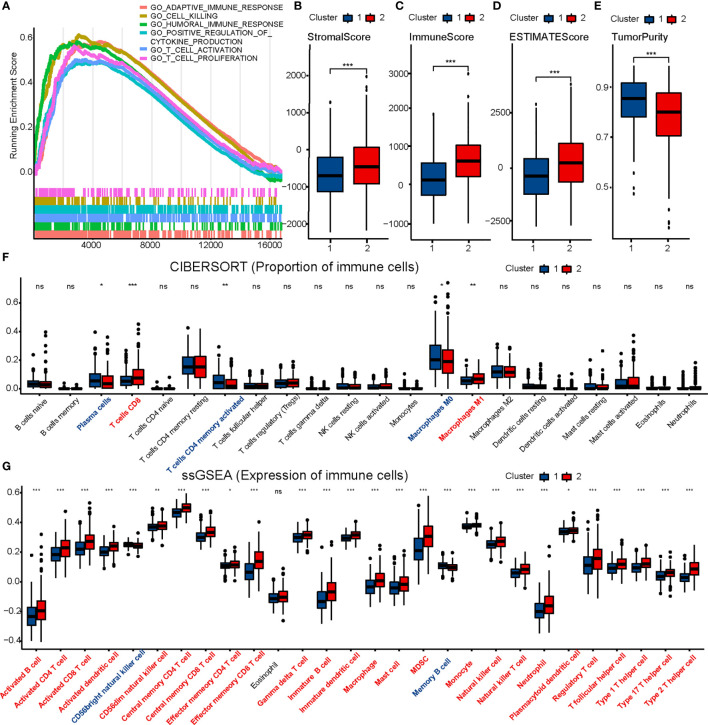
Comparison of immune characteristics between two clusters. Comparison of functional enrichment **(A)**, stromal score **(B)**, immune score **(C)**, ESTIMATE score **(D)**, tumor purity **(E)**, proportion of immune cells **(F)** and expression of immune cells **(G)** between two clusters. The P values are labeled using asterisks (ns, no significance, *P < 0.05, **P < 0.01, ***P < 0.001).

GSEA was performed to understand the functional differences between the two clusters. All differentially expressed genes (Cluster 2 *vs.* Cluster 1) were included in the GSEA. We identified many significant pathways related to immunity in the enrichment of MSigDB Collection (c5.cp.v7.0.symbols.gmt), including adaptive immune response, cell killing, humoral immune response, positive regulation of cytokine production, T cell activation, and T cell proliferation (**Figure 2A**).

### Comparison of Immune Infiltration

ESTIMATE, CIBERSORT, and ssGSEA were performed to understand the differences in immunological function better. Cluster 2 had higher stromal, immune, and ESTIMATE scores, and lower tumor purity than Cluster 1 in the ESTIMATE analysis (**Figures 2B–E**). Furthermore, CIBERSORT analysis demonstrated that Cluster 2 to have a higher proportion of CD8 T cells (**Figure 2F**). ssGSEA showed 25 immune cell subtypes (such as activated B cells, activated CD4 T cells, activated CD8 T cells, activated dendritic cells, natural killer cells, and natural killer T cells) to be highly expressed in Cluster 2 (**Figure 2G**). A heatmap was prepared to show the overall conditions of the 28 immune cell subtypes in the two clusters (**Supplementary Figure 4**). Results indicated that Cluster 2 tended to have a stronger immune infiltration than Cluster 1, especially regarding CD8 T cells.

### Evaluation of Sensitivity to Immunotherapy

**Figure 3:**
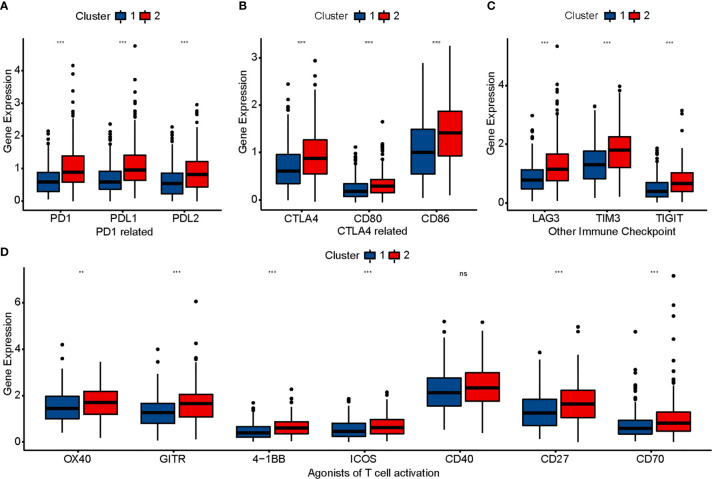
Comparison of immunomodulatory drugs’ targets in clinical trials for metastatic colorectal cancer between two clusters. The P values are labeled using asterisks (ns, no significance, **P < 0.01, ***P < 0.001).

To evaluate the sensitivity of patients with COAD to immunotherapy, we identified some targets of immunomodulatory drugs in clinical trials for metastatic colorectal cancer. We then compared the expression of these immunomodulatory targets between the two clusters and found most of the immunomodulatory targets (PD1, PDL1, PDL2, CTLA4, CD80, CD86, LAG3, TIM3, TIGHT, OX40, GITR, 4-1BB, ICOS, CD27, and CD70) to be expressed significantly higher in Cluster 2 (**Figures 3A–D**). The results suggested that Cluster 2 may show better response to immunotherapy than Cluster 1.

### Comparison of Genetic Mutation

**Figure 4:**
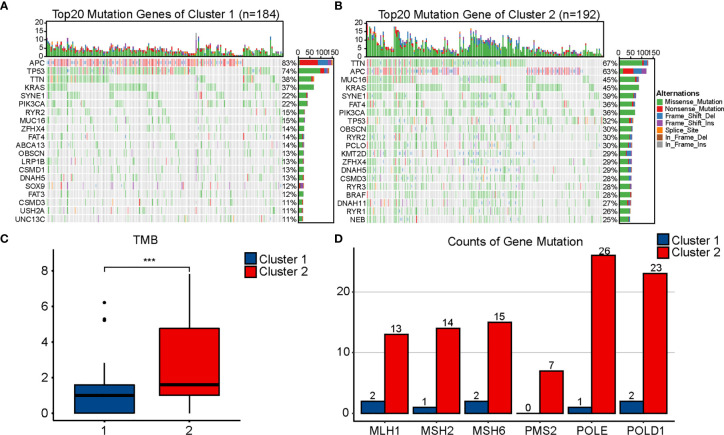
Comparison of mutational landscapes between two clusters. Mutational landscape of Cluster 1 **(A)** and Cluster 2 **(B)**. **(C)** Comparison of tumor mutation burden (TMB) between two clusters. **(D)** Comparison of gene mutation related to mismatch repair and POLE proofreading domain between two clusters. The P values are labeled using asterisks (***P < 0.001).

Different genetic mutations can influence the efficacy of immunotherapy differently; therefore, we evaluated the mutational conditions in COAD. Landscapes of mutation profiles between the two clusters are shown in **Figures 4A, B**. Cluster 2 had higher TMB and more numbers of MLH1, MSH2, MSH6, PMS2, POLE, and POLD1 mutations than Cluster 1 (**Figures 4C, D**), which once again indicated that the effect of immunotherapy may be better in Cluster 2.

### WGCNA and Identification of Hub Genes Related With m6A and Immunity

**Figure 5:**
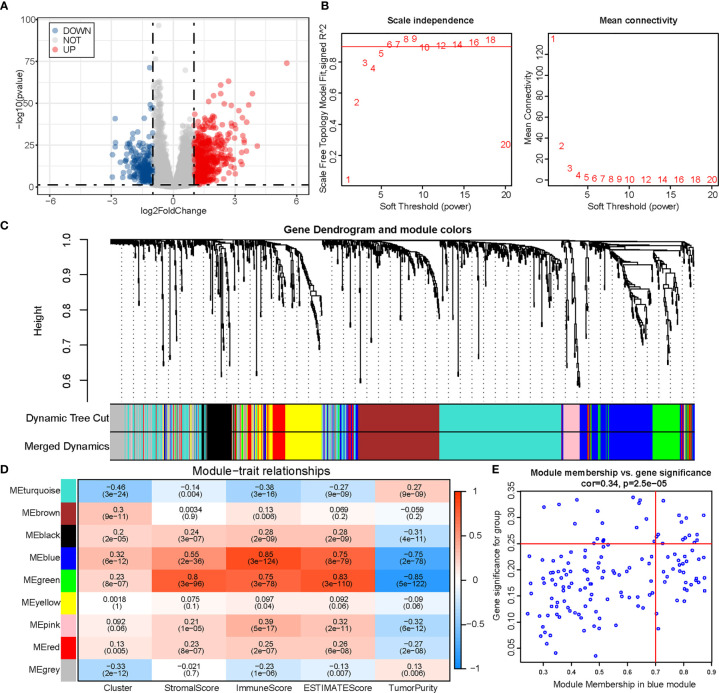
Identification of module genes associated with both clustering and immunity in the WGCNA. **(A)** Volcano plot of differential analysis. **(B)** Analysis of network topology for soft powers. **(C)** Gene dendrogram and module colors. **(D)** Heatmap between module eigengenes and cluster, ESTIMATE results. **(E)** Scatter plot of module eigengenes in the blue module.

We obtained 978 DEGs (721 upregulated and 257 downregulated) between the two clusters, and results were visualized using a volcano plot (**Figure 5A**). The genes were then considered for the WGCNA (**Figures 5B, C**). To identify a module related to both m6A and immunity, we performed a correlation between modules and traits (**Figure 5D**). The blue module was selected owing to its correlation with m6A (R = 0.32, P = 6e-12) and immunity (R = 0.85, P = 3e-124). Thereafter, we obtained 14 hub genes (WARS, SLC2A5, UBASH3B, NKG7, GNLY, GZMH, LAG3, GZMA, HAPLN3, CTSW, PDCD1, CCL4, RARRES3, and KIR2DL4) from the blue module based on MM >0.7 and GS >0.25 (**Figure 5E**).

### Functional Enrichment of Hub Genes and Their Correlation With Immune Infiltration

**Figure 6:**
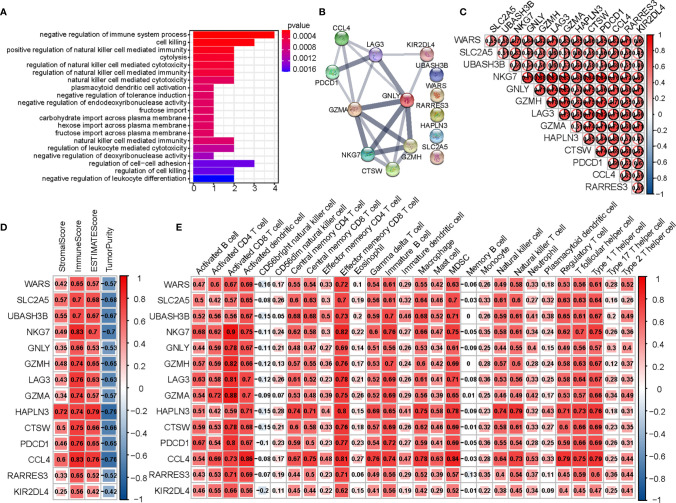
Analysis of 14 hub genes. **(A)** The GO analysis of hub genes. **(B)** PPI network of hub genes. **(C)** Correlation between hub genes. **(D)** Correlation between hub genes and results of ESTIMATE. **(E)** Correlation between hub genes and expression of immune cells (ssGSEA).

To determine the biochemical functions of the 14 hub genes, we performed a GO enrichment analysis (**Figure 6A**). The most significant GO term was negative regulation of immune system. We also conducted PPI analysis and checked correlations across the genes (**Figures 6B, C**). Spearman’s correlation analysis between genes and immune infiltration (ESTIMATE and ssGSEA) showed most of the genes to be significantly correlated with immunity (**Figures 6D, E**).

### GEO Validation of Immune Characteristics Between Two Clusters

**Figure 7:**
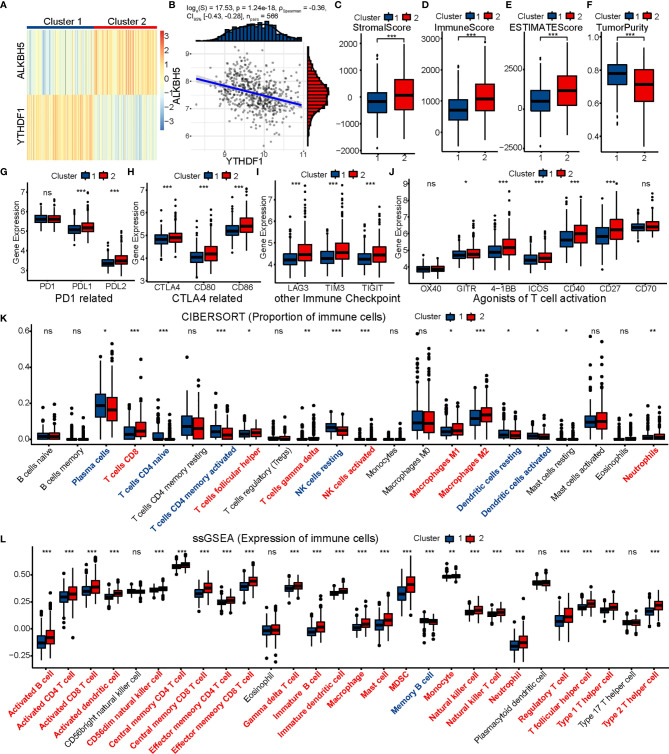
GSE39582 validation of immune contexture between two clusters. **(A)** GSE39582 patients are divided into two clusters according to ALKBH5 and YTHDF1. **(B)** Association between ALKBH5 and YTHDF1 expression in GSE39582. **(C–K)** Comparison of stromal score **(C)**, immune score **(D)**, ESTIMATE score **(E)**, tumor purity **(F)**, targets of immunomodulatory drugs **(G**–**J)**, proportion of immune cells **(K)** and expression of immune cells **(L)** between two clusters. The P values are labeled using asterisks (ns, no significance, *P < 0.05, **P < 0.01, ***P < 0.001).

First, we divided 566 colon cancer samples from GSE39582 into two clusters in the same way as performed in TCGA (**Figure 7A**) and found the distribution of ALKBH5 and YTHDF1 expression in the two clusters to be quite similar to that in TCGA. We also calculated the Spearman’s correlation coefficient for ALKBH5 and YTHDF1 (R = −0.36, P = 1.24e-18) (**Figure 7B**). The expression of immunomodulatory targets and extent of immune infiltration (ESTIMATE, CIBERSORT, and ssGSEA) were evaluated in the same manner (**Figures 7C–L**). Cluster 2 was clearly more active regarding the immune system than Cluster 1.

### Verification of Expression Levels of ALKBH5 and YTHDF1 in CRC and Adjacent Tissues

**Figure 8:**
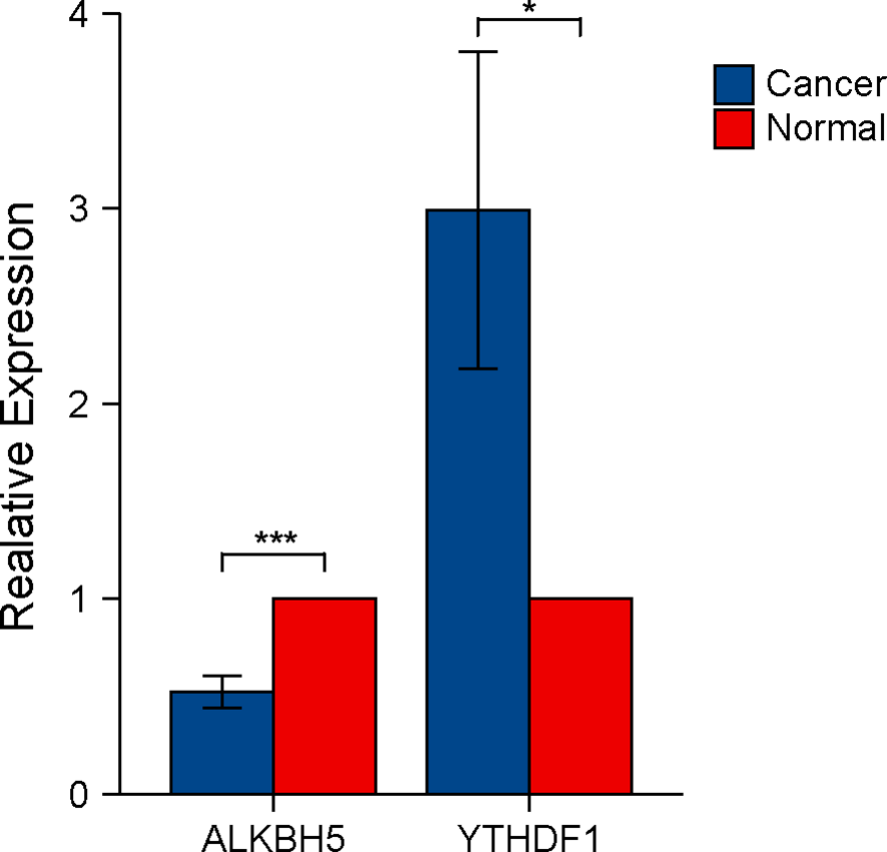
Verification of ALKBH5 and YTHDF1 expression in CRC tissues using RT-qPCR. The P values are labeled using asterisks (ns, no significance, *P < 0.05, ***P < 0.001).

We tested the expression levels of ALKBH5 and YTHDF1 in 12 CRC tissues and paired adjacent tissues using RT-qPCR. Results indicated CRC tissues to have lower expression of ALKBH5 and higher expression of YTHDF1 than the paired adjacent tissues (**Figure 8**).

## Discussion

ALKBH5 is expressed at low levels in colon cancer; its overexpression inhibits cell metastasis *in vivo* and cell invasion *in vitro*, thus suggesting it as a tumor suppressor (34). A recent study has reported that the expression and function of ALKBH5 in different types of cancer are variable (35). Although ALKBH5 has been proven to correlate with the response to anti-PD1 therapy in melanoma, the association between ALKBH5 expression and response to immunotherapy in patients with COAD still remains unclear (15). YTHDF1 is highly expressed and enhances stem cell-like activity in CRC (36). The high expression of YTHDF1 could lead to low immune cell abundance, since high stemness indices represent a low immune cell fraction and low PD-L1 expression (37). The expression of ALKBH5 and YTHDF1 in patients with COAD in our study was consistent with those in previous studies. In addition, we found a negative correlation between ALKBH5 and YTHDF1, suggesting that their functions may have a cross-talk or interaction upon m6A modification. A previous study had reported that ALKBH5 suppresses tumor progression in non-small cell lung cancer in a YTHDF1-dependent manner (38). Moreover, their relevance to the immune score in the ESTIMATE analysis was just the opposite. Therefore, both ALKBH5 and YTHDF1 may participate in the regulation of m6A modification, which can in turn influence immune infiltration and response to immunotherapy in patients with COAD.

We applied consensus clustering to divide patients with COAD from TCGA into two clusters: Cluster 1 (ALKBH5: low expression; YTHDF1: high expression) and Cluster 2 (ALKBH5: high expression; YTHDF1: low expression). Moreover, we investigated the relationship between the two clusters and the CMS. We found CMS2 to be mostly classified into Cluster 1, whereas CMS1 and CMS3 were mostly classified into Cluster 2. There was no difference in CMS4. CMS1 represented microsatellite instability immune type with hypermutation, MSI, and strong immune activation; CMS2 represented canonical type with remarkable WNT and MYC activation; CMS3 represented metabolic type with epithelial and evident metabolic dysregulation (29). From the perspective of CMS, we inferred Cluster 2 to possibly possess more immune infiltration than Cluster 1. We then evaluated their clinical characteristics and discovered Cluster 1 to have a higher N stage than Cluster 2, which may have resulted from the inhibition of metastasis by ALKBH5.

To further investigate the functional differences between the two clusters, we used TCGA-COAD data for GSEA and found some immune-related pathways, such as adaptive immune response, cell killing, cytokine production, and T cell activation to be enriched in Cluster 2. This suggested that Cluster 2 may act more actively in immune response than Cluster 1.

Next, we compared the immune characteristics of the two clusters using the ESTIMATE, CIBERSORT, and ssGSEA methods. In the ESTIMATE analysis, Cluster 2 was proven to possess higher stromal, immune, and ESTIMATE scores than Cluster 1, thereby implying that Cluster 2 had a vibrant tumor immune microenvironment. In the CIBERSORT analysis, we found the proportion of CD8 T cells and M1 subtype macrophages to be significantly elevated in Cluster 2. Previous studies had demonstrated CD8 T cells to have the strongest effect on patient prognosis in most tumor-infiltrating immune cell subtypes (39). In the ssGSEA analysis, 25 immune cell subtypes showed significantly higher expression in Cluster 2, including CD8 T cells, T helper cells (CD4), dendritic cells (DCs), natural killer (NK) cells, natural killer T (NKT) cells, and macrophages. Tumor-infiltrating T cells have a major impact on the clinical attributes of CRC. High infiltration of CD8 T cells can predict the response to drugs and improve survival in patients with CRC and hepatic metastases (40). A previous study had illustrated that patients with high expression of Th1 have a prolonged prognosis, whereas those with high expression of Th17 have poor prognosis in CRC. In addition, the effect of Th1 seemed to surpass the effect of Th17 on survival (41). DCs have been reported as key antigen-presenting cells that promote anti-tumor immunity by activating T cells (42). Moreover, conventional type 1 dendritic cells are known to be recruited into the tumor microenvironment following stimulation by NK cells (43). The latter have cytotoxic capacity in anti-tumor immunity, and their extensive infiltration leads to a favorable outcome in CRC (44). NKT cells could reinvigorate the exhausted CD8 T cells in an anti-PD-1-resistant tumor model, hence playing a pivotal role in anti-tumor immunity (45). A previous study had shown that high NKT cell infiltration to be an independent favorable prognostic factor in CRC (46). Macrophages are conventionally divided into M1 (proinflammatory; anti-tumor) and M2 (anti-inflammatory; tumor-promoting) subtypes. According to the results of CIBERSORT analysis, Cluster 2 had a higher proportion of M1 subtype macrophages than Cluster 1, which suggested that Cluster 2 could easily achieve anti-tumor Th1-type responses while Cluster 1 tended to establish a tolerogenic microenvironment (47). Based on our study of immune contexture, Cluster 2 had more extensive immune cell infiltration than Cluster 1. Therefore, Cluster 2 may have more immunological competence and be more likely to benefit from immunotherapy.

Previous studies reported that programmed cell death 1 (PD1), programmed cell death 1 ligand 1 (PDL1), and cytotoxic T lymphocyte antigen 4 (CTLA4) are approved as targets of immune checkpoint inhibitors (ICIs) by the FDA (48). In addition, lymphocyte activation gene-3 (LAG3), T cell immunoglobulin-3 (TIM3), and T cell immunoglobulin and ITIM domain (TIGIT) are regarded as co-inhibitory receptor targets (49). In this study, we compared two clusters of immunomodulatory drugs, which have been included in clinical trials for metastatic CRC (48). Most of these targets were found to be significantly high in expression in Cluster 2.

Next, we analyzed the mutational landscapes of the two clusters, and found a remarkable difference between them. We found Cluster 2 to have more TMB than Cluster 1. TMB may affect the generation of immunogenic peptides and thereby influence the response to immunotherapy (50). Furthermore, CRC can be categorized into two groups based on microsatellite instability (MSI) and mismatch repair (MMR), namely dMMR-MSI-H and pMMR-MSI-L. The signature of dMMR-MSI-H in patients with CRC is a specific biomarker for evaluating the response to immunotherapy. In addition to the hypermutation caused by dMMR-MSI-H, POLE proofreading domain mutation also leads to a remarkable hypermutation, which may result in excellent prognosis (48). Our study suggested that Cluster 2 possesses a higher mutation rate of MMR-related genes (MLH1, MSH2, MSH6, and PMS2) and POLE/POLD1 than Cluster 1, hence implying that Cluster 2 would show better effect of immunotherapy.

We next performed WGCNA to identify the blue module that differed between the two clusters in relation to both ALKBH5/YTHDF1 and immune score. Based on MM and GS, we obtained 14 genes from this module, including PD1 (PDCD1) and LAG3. An explicit synergistic interaction between PD1 and LAG3 has already been reported, and they have been shown to mediate T cell exhaustion together (51); this probably occurs in Cluster 2. Therefore, we speculated that Cluster 2 could acquire a better response to immunotherapy than Cluster 1 by evaluating the extent of infiltration extent of immune cells, expression of ICIs, and mutational burden; this difference might result from m6A modification mediated by ALKBH5 and YTHDF1.

Based on the immunological features, Cluster 2 was considered to have a hot tumor, whereas Cluster 1 tended to be have a cold tumor (52). Since our RT-qPCR results verified the expression of ALKBH5 and YTHDF1 in 12 pairs of cancer and normal tissues, we inferred Cluster 1 to represent the overall immunological characteristics of COAD, thus showing poor immune response. ALKBH5 and YTHDF1 could possibly play a potential role in the transformation of cold to hot tumor in COAD.

Although the comprehensive analysis improved our understanding of the relationship between ALKBH5/YTHDF1 and immunity, and we used 566 patients with GSE39582 as the external validation set, there are still some limitations in the current study. First, it was a retrospective study. Therefore, a prospective study should be conducted in future in order to avoid analysis bias associated with retrospective studies. Moreover, the study was performed based on TCGA and GEO; we could not illustrate the expression of ALKBH5 and YTHDF1 from the protein level or demonstrate the direct mechanisms of ALKBH5/YTHDF1 in anti-tumor immunity. So further studies to unravel the direct mechanisms should be performed.

## Conclusion

By clustering patients of TCGA-COAD and GSE39582 based on the expression of ALKBH5 and YTHDF1, we demonstrated Cluster 2 (ALKBH5: highly expressed; YTHDF1: lowly expressed) to have more infiltration of immune cells, expression of ICI targets, TMB, and proportion of dMMR-MSI-H than Cluster 1 (ALKBH5: lowly expressed; YTHDF1: highly expressed), thereby suggesting that Cluster 2 acquired better response to immunotherapy. Our findings illustrated that ALKBH5 and YTHDF1 have potential in tumor immunity and provided novel insights into the relationship between m6A modification and immunity.

## Data Availability Statement

The original contributions presented in the study are included in the article/**Supplementary Material**. Further inquiries can be directed to the corresponding authors.

## Ethics Statement

The studies involving human participants were reviewed and approved by the institutional ethics board of the First Hospital of China Medical University. The patients/participants provided their written informed consent to participate in this study.

## Author Contributions

GY designed the study and wrote the manuscript. YA performed the literature search and collected data for the manuscript. BX analyzed the data. NW edited the figures and tables. MS and XS revised the manuscript. All authors contributed to the article and approved the submitted version.

## Funding

This work was supported by the National Natural Science Foundation of China (81670506).

## Conflict of Interest

The authors declare that the research was conducted in the absence of any commercial or financial relationships that could be construed as a potential conflict of interest.

## Supplementary Material

Supplementary Figure 1 Heatmap corresponding to the consensus matrix for k = 2 using consensus clustering.

Supplementary Figure 2 Relationship between the two clusters and the consensus molecular subtypes (CMS).

Supplementary Figure 3 Survival analysis of 2 clusters.

Supplementary Figure 4 Heatmap shows overall condition of 28 immune cells subtypes between 2 clusters.

## References

[1] Sung H Ferlay J Siegel RL Laversanne M Soerjomataram I Jemal A. Global Cancer Statistics 2020: GLOBOCAN Estimates of Incidence and Mortality Worldwide for 36 Cancers in 185 Countries. CA Cancer J Clin (2021) 0:1–41. 10.3322/caac.21660 33538338

[2] Lombardi L Morelli F Cinieri S Santini D Silvestris N Fazio N. Adjuvant Colon Cancer Chemotherapy: Where We Are and Where We’ll Go. Cancer Treat Rev (2010) 36 Suppl 3:S34–41. 10.1016/S0305-7372(10)70018-9 21129608

[3] Mattiuzzi C Sanchis-Gomar F Lippi G. Concise Update on Colorectal Cancer Epidemiology. Ann Transl Med (2019) 7(21):609. 10.21037/atm.2019.07.91 32047770 PMC7011596

[4] Mutch MG. Molecular Profiling and Risk Stratification of Adenocarcinoma of the Colon. J Surg Oncol (2007) 96(8):693–703. 10.1002/jso.20915 18081153

[5] Riaz N Morris L Havel JJ Makarov V Desrichard A Chan TA. The Role of Neoantigens in Response to Immune Checkpoint Blockade. Int Immunol (2016) 28(8):411–9. 10.1093/intimm/dxw019 PMC4986233 27048318

[6] Chalabi M Fanchi LF Dijkstra KK Van den Berg JG Aalbers AG Sikorska K. Neoadjuvant Immunotherapy Leads to Pathological Responses in MMR-proficient and MMR-deficient Early-Stage Colon Cancers. Nat Med (2020) 26(4):566–76. 10.1038/s41591-020-0805-8 32251400

[7] Diaz LA Jr Le DT. PD-1 Blockade in Tumors With Mismatch-Repair Deficiency. N Engl J Med (2015) 373(20):1979. 10.1056/NEJMc1510353 26559582

[8] Yang D Qiao J Wang G Lan Y Li G Guo X. N6-Methyladenosine Modification of lincRNA 1281 is Critically Required for mESC Differentiation Potential. Nucleic Acids Res (2018) 46(8):3906–20. 10.1093/nar/gky130 PMC5934679 29529255

[9] Zhou C Molinie B Daneshvar K Pondick JV Wang J Van Wittenberghe N. Genome-Wide Maps of m6A CircRNAs Identify Widespread and Cell-Type-Specific Methylation Patterns That Are Distinct From Mrnas. Cell Rep (2017) 20(9):2262–76. 10.1016/j.celrep.2017.08.027 PMC5705222 28854373

[10] Alarcon CR Lee H Goodarzi H Halberg N Tavazoie SF. N6-Methyladenosine Marks Primary microRNAs for Processing. Nature (2015) 519(7544):482–5. 10.1038/nature14281 PMC4475635 25799998

[11] Desrosiers R Friderici K Rottman F. Identification of Methylated Nucleosides in Messenger RNA From Novikoff Hepatoma Cells. Proc Natl Acad Sci USA (1974) 71(10):3971–5. 10.1073/pnas.71.10.3971 PMC434308 4372599

[12] Nombela P Miguel-Lopez B Blanco S. The Role of M(6)a, M(5)C and Psi RNA Modifications in Cancer: Novel Therapeutic Opportunities. Mol Cancer (2021) 20(1):18. 10.1186/s12943-020-01263-w 33461542 PMC7812662

[13] Huang H Weng H Chen J. M(6)a Modification in Coding and Non-coding RNAs: Roles and Therapeutic Implications in Cancer. Cancer Cell (2020) 37(3):270–88. 10.1016/j.ccell.2020.02.004 PMC7141420 32183948

[14] Zheng G Dahl JA Niu Y Fedorcsak P Huang CM Li CJ. ALKBH5 is a Mammalian RNA Demethylase That Impacts RNA Metabolism and Mouse Fertility. Mol Cell (2013) 49(1):18–29. 10.1016/j.molcel.2012.10.015 23177736 PMC3646334

[15] Li N Kang Y Wang L Huff S Tang R Hui H. ALKBH5 Regulates anti-PD-1 Therapy Response by Modulating Lactate and Suppressive Immune Cell Accumulation in Tumor Microenvironment. Proc Natl Acad Sci USA (2020) 117(33):20159–70. 10.1073/pnas.1918986117 PMC7443867 32747553

[16] Tang B Yang Y Kang M Wang Y Wang Y Bi Y. M(6)a Demethylase ALKBH5 Inhibits Pancreatic Cancer Tumorigenesis by Decreasing WIF-1 RNA Methylation and Mediating Wnt Signaling. Mol Cancer (2020) 19(1):3. 10.1186/s12943-019-1128-6 31906946 PMC6943907

[17] Wang X Lu Z Gomez A Hon GC Yue Y Han D. N6-Methyladenosine-Dependent Regulation of Messenger RNA Stability. Nature (2014) 505(7481):117–20. 10.1038/nature12730 PMC3877715 24284625

[18] Han D Liu J Chen C Dong L Liu Y Chang R. Anti-Tumour Immunity Controlled Through mRNA M(6)a Methylation and YTHDF1 in Dendritic Cells. Nature (2019) 566(7743):270–4. 10.1038/s41586-019-0916-x PMC6522227 30728504

[19] Colaprico A Silva TC Olsen C Garofano L Cava C Garolini D. TCGAbiolinks: An R/Bioconductor Package for Integrative Analysis of TCGA Data. Nucleic Acids Res (2016) 44(8):e71. 10.1093/nar/gkv1507 26704973 PMC4856967

[20] Mayakonda A Lin DC Assenov Y Plass C Koeffler HP. Maftools: Efficient and Comprehensive Analysis of Somatic Variants in Cancer. Genome Res (2018) 28(11):1747–56. 10.1101/gr.239244.118 PMC6211645 30341162

[21] Gu Z Eils R Schlesner M. Complex Heatmaps Reveal Patterns and Correlations in Multidimensional Genomic Data. Bioinformatics (2016) 32(18):2847–9. 10.1093/bioinformatics/btw313 27207943

[22] Marisa L de Reynies A Duval A Selves J Gaub MP Vescovo L. Gene Expression Classification of Colon Cancer Into Molecular Subtypes: Characterization, Validation, and Prognostic Value. PLoS Med (2013) 10(5):e1001453. 10.1371/journal.pmed.1001453 23700391 PMC3660251

[23] Yoshihara K Shahmoradgoli M Martinez E Vegesna R Kim H Torres-Garcia W. Inferring Tumour Purity and Stromal and Immune Cell Admixture From Expression Data. Nat Commun (2013) 4:2612. 10.1038/ncomms3612 24113773 PMC3826632

[24] Newman AM Liu CL Green MR Gentles AJ Feng W Xu Y. Robust Enumeration of Cell Subsets From Tissue Expression Profiles. Nat Methods (2015) 12(5):453–7. 10.1038/nmeth.3337 PMC4739640 25822800

[25] Hanzelmann S Castelo R Guinney J. GSVA: Gene Set Variation Analysis for Microarray and RNA-seq Data. BMC Bioinformatics (2013) 14:7. 10.1186/1471-2105-14-7 23323831 PMC3618321

[26] Bindea G Mlecnik B Tosolini M Kirilovsky A Waldner M Obenauf AC. Spatiotemporal Dynamics of Intratumoral Immune Cells Reveal the Immune Landscape in Human Cancer. Immunity (2013) 39(4):782–95. 10.1016/j.immuni.2013.10.003 24138885

[27] Wilkerson MD Hayes DN. ConsensusClusterPlus: A Class Discovery Tool With Confidence Assessments and Item Tracking. Bioinformatics (2010) 26(12):1572–3. 10.1093/bioinformatics/btq170 PMC2881355 20427518

[28] Eide PW Bruun J Lothe RA Sveen A. CMScaller: An R Package for Consensus Molecular Subtyping of Colorectal Cancer Pre-Clinical Models. Sci Rep (2017) 7(1):16618. 10.1038/s41598-017-16747-x 29192179 PMC5709354

[29] Guinney J Dienstmann R Wang X de Reynies A Schlicker A Soneson C. The Consensus Molecular Subtypes of Colorectal Cancer. Nat Med (2015) 21(11):1350–6. 10.1038/nm.3967 PMC4636487 26457759

[30] Yu G Wang LG Han Y He QY. clusterProfiler: An R Package for Comparing Biological Themes Among Gene Clusters. OMICS (2012) 16(5):284–7. 10.1089/omi.2011.0118 PMC3339379 22455463

[31] Love MI Huber W Anders S. Moderated Estimation of Fold Change and Dispersion for RNA-seq Data With DESeq2. Genome Biol (2014) 15(12):550. 10.1186/s13059-014-0550-8 25516281 PMC4302049

[32] Langfelder P Horvath S. WGCNA: An R Package for Weighted Correlation Network Analysis. BMC Bioinformatics (2008) 9:559. 10.1186/1471-2105-9-559 19114008 PMC2631488

[33] Szklarczyk D Gable AL Lyon D Junge A Wyder S Huerta-Cepas J. STRING v11: Protein-Protein Association Networks With Increased Coverage, Supporting Functional Discovery in Genome-Wide Experimental Datasets. Nucleic Acids Res (2019) 47(D1):D607–D13. 10.1093/nar/gky1131 PMC6323986 30476243

[34] Yang P Wang Q Liu A Zhu J Feng J. ALKBH5 Holds Prognostic Values and Inhibits the Metastasis of Colon Cancer. Pathol Oncol Res (2020) 26(3):1615–23. 10.1007/s12253-019-00737-7 31506804

[35] Wang J Wang J Gu Q Ma Y Yang Y Zhu J. The Biological Function of m6A Demethylase ALKBH5 and its Role in Human Disease. Cancer Cell Int (2020) 20:347. 10.1186/s12935-020-01450-1 32742194 PMC7388453

[36] Bai Y Yang C Wu R Huang L Song S Li W. YTHDF1 Regulates Tumorigenicity and Cancer Stem Cell-Like Activity in Human Colorectal Carcinoma. Front Oncol (2019) 9:332. 10.3389/fonc.2019.00332 31131257 PMC6509179

[37] Malta TM Sokolov A Gentles AJ Burzykowski T Poisson L Weinstein JN. Machine Learning Identifies Stemness Features Associated With Oncogenic Dedifferentiation. Cell (2018) 173(2):338–54.e15. 10.1016/j.cell.2018.03.034 29625051 PMC5902191

[38] Jin D Guo J Wu Y Yang L Wang X Du J. M(6)a Demethylase ALKBH5 Inhibits Tumor Growth and Metastasis by Reducing YTHDFs-mediated YAP Expression and Inhibiting Mir-107/LATS2-Mediated YAP Activity in NSCLC. Mol Cancer (2020) 19(1):40. 10.1186/s12943-020-01161-1 32106857 PMC7045432

[39] Bruni D Angell HK Galon J. The Immune Contexture and Immunoscore in Cancer Prognosis and Therapeutic Efficacy. Nat Rev Cancer (2020) 20(11):662–80. 10.1038/s41568-020-0285-7 32753728

[40] Galon J Costes A Sanchez-Cabo F Kirilovsky A Mlecnik B Lagorce-Pages C. Type, Density, and Location of Immune Cells Within Human Colorectal Tumors Predict Clinical Outcome. Science (2006) 313(5795):1960–4. 10.1126/science.1129139 17008531

[41] Tosolini M Kirilovsky A Mlecnik B Fredriksen T Mauger S Bindea G. Clinical Impact of Different Classes of Infiltrating T Cytotoxic and Helper Cells (Th1, th2, Treg, th17) in Patients With Colorectal Cancer. Cancer Res (2011) 71(4):1263–71. 10.1158/0008-5472.CAN-10-2907 21303976

[42] Wculek SK Cueto FJ Mujal AM Melero I Krummel MF Sancho D. Dendritic Cells in Cancer Immunology and Immunotherapy. Nat Rev Immunol (2020) 20(1):7–24. 10.1038/s41577-019-0210-z 31467405

[43] Bottcher JP Bonavita E Chakravarty P Blees H Cabeza-Cabrerizo M Sammicheli S. NK Cells Stimulate Recruitment of cDC1 Into the Tumor Microenvironment Promoting Cancer Immune Control. Cell (2018) 172(5):1022–37.e14. 10.1016/j.cell.2018.01.004 29429633 PMC5847168

[44] Coca S Perez-Piqueras J Martinez D Colmenarejo A Saez MA Vallejo C. The Prognostic Significance of Intratumoral Natural Killer Cells in Patients With Colorectal Carcinoma. Cancer (1997) 79(12):2320–8. 10.1002/(sici)1097-0142(19970615)79:12<2320::aid-cncr5>3.0.co;2-p 9191519

[45] Bae EA Seo H Kim BS Choi J Jeon I Shin KS. Activation of NKT Cells in an Anti-PD-1-Resistant Tumor Model Enhances Antitumor Immunity by Reinvigorating Exhausted CD8 T Cells. Cancer Res (2018) 78(18):5315–26. 10.1158/0008-5472.CAN-18-0734 30012672

[46] Tachibana T Onodera H Tsuruyama T Mori A Nagayama S Hiai H. Increased Intratumor Valpha24-positive Natural Killer T Cells: A Prognostic Factor for Primary Colorectal Carcinomas. Clin Cancer Res (2005) 11(20):7322–7. 10.1158/1078-0432.CCR-05-0877 16243803

[47] Aras S Zaidi MR. TAMeless Traitors: Macrophages in Cancer Progression and Metastasis. Br J Cancer (2017) 117(11):1583–91. 10.1038/bjc.2017.356 PMC5729447 29065107

[48] Ganesh K Stadler ZK Cercek A Mendelsohn RB Shia J Segal NH. Immunotherapy in Colorectal Cancer: Rationale, Challenges and Potential. Nat Rev Gastroenterol Hepatol (2019) 16(6):361–75. 10.1038/s41575-019-0126-x PMC7295073 30886395

[49] Anderson AC Joller N Kuchroo VK. Lag-3, Tim-3, and TIGIT: Co-Inhibitory Receptors With Specialized Functions in Immune Regulation. Immunity (2016) 44(5):989–1004. 10.1016/j.immuni.2016.05.001 27192565 PMC4942846

[50] Havel JJ Chowell D Chan TA. The Evolving Landscape of Biomarkers for Checkpoint Inhibitor Immunotherapy. Nat Rev Cancer (2019) 19(3):133–50. 10.1038/s41568-019-0116-x PMC6705396 30755690

[51] Andrews LP Marciscano AE Drake CG Vignali DA. LAG3 (CD223) as a Cancer Immunotherapy Target. Immunol Rev (2017) 276(1):80–96. 10.1111/imr.12519 28258692 PMC5338468

[52] Galon J Bruni D. Approaches to Treat Immune Hot, Altered and Cold Tumours With Combination Immunotherapies. Nat Rev Drug Discov (2019) 18(3):197–218. 10.1038/s41573-018-0007-y 30610226

