# Supplementary figures and images for: Potential Impact of ALKBH5 and YTHDF1 on Tumor Immunity in Colon Adenocarcinoma
Source: Front Oncol. 2021 May 17;11:670490. doi: 10.3389/fonc.2021.670490 (PMC8165310; doi:10.3389/fonc.2021.670490)

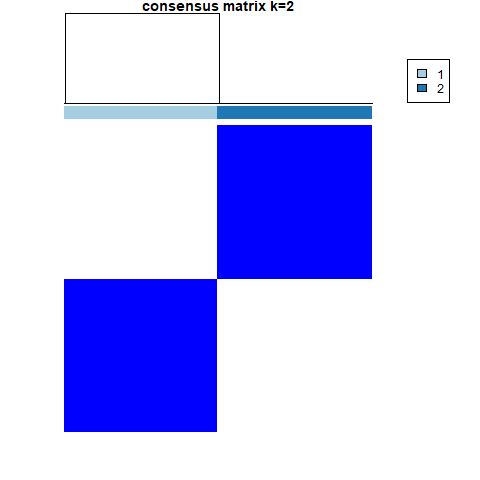

Supplement: Supplementary Figure 1 — Heatmap corresponding to the consensus matrix for k = 2 using consensus clustering. [file Image_1.png]

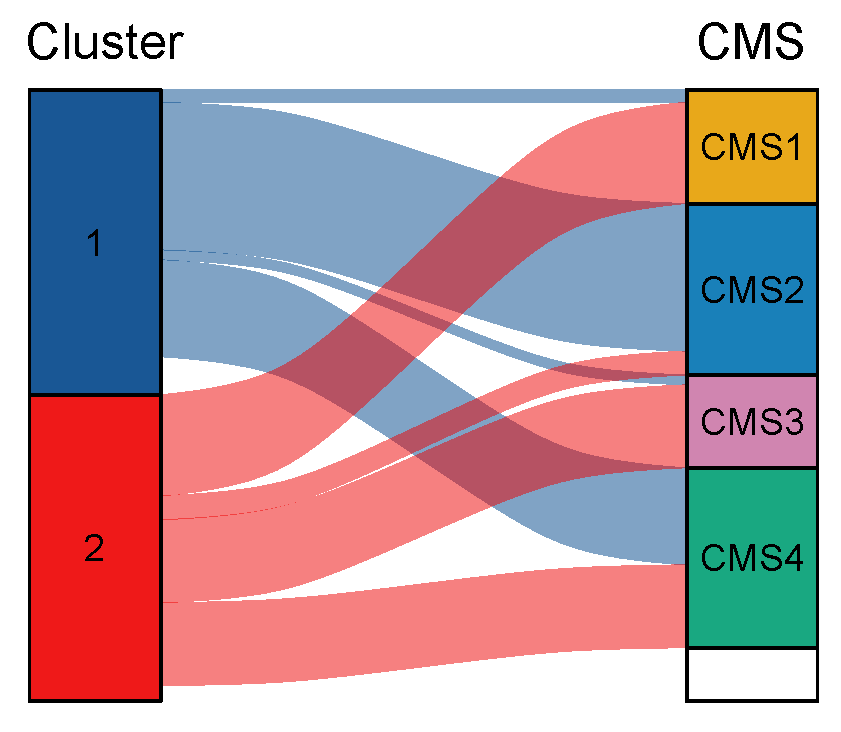

Supplement: Supplementary Figure 2 — Relationship between the two clusters and the consensus molecular subtypes (CMS). [file Image_2.tif]

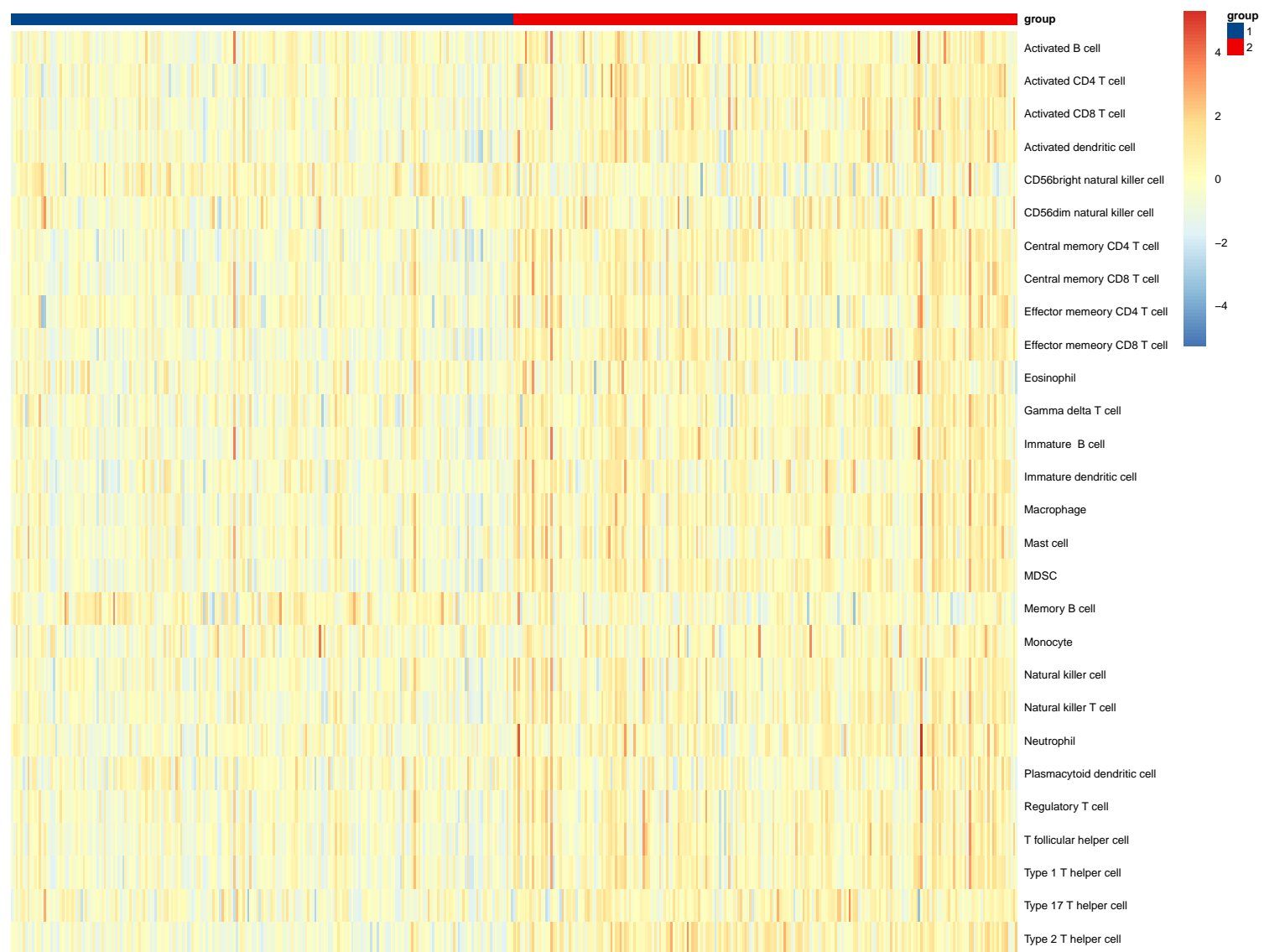

Supplement: Supplementary Figure 4 — Heatmap shows overall condition of 28 immune cells subtypes between 2 clusters. [file Image_4.pdf]
