# Supplementary material for: Potential Impact of ALKBH5 and YTHDF1 on Tumor Immunity in Colon Adenocarcinoma
Source: Front Oncol. 2021 May 17;11:670490. doi: 10.3389/fonc.2021.670490 (PMC8165310; doi:10.3389/fonc.2021.670490)
Supplement: Supplementary Figure 3 — Survival analysis of 2 clusters. [file Image_3.pdf]

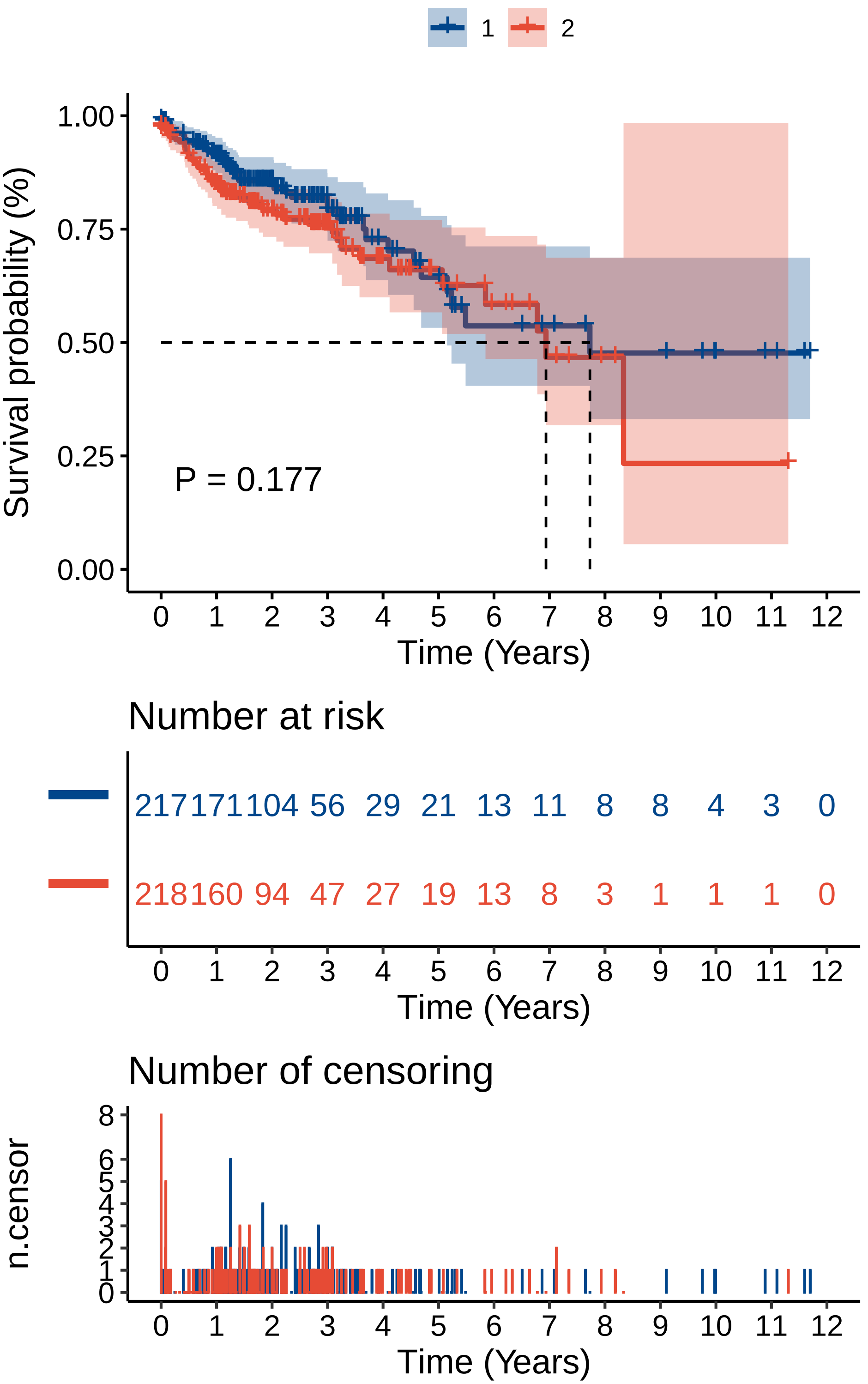
1
2
Survival probability (%)
1.00
0.75
0.50
0.25
0.00
P = 0.177
0 1 2 3 4 5 6 7 8 9 10 11 12
Time (Years)
Number at risk
217 171 104 56 29 21 13 11 8 8 4 3 0
218 160 94 47 27 19 13 8 3 1 1 1 0
0 1 2 3 4 5 6 7 8 9 10 11 12
Time (Years)
Number of censoring
n.censor
8
6
5
4
3
2
1
0
0 1 2 3 4 5 6 7 8 9 10 11 12
Time (Years)
